# A New Total Digital Smile Planning Technique (3D-DSP) to Fabricate CAD-CAM Mockups for Esthetic Crowns and Veneers

**DOI:** 10.1155/2016/6282587

**Published:** 2016-07-10

**Authors:** F. Cattoni, F. Mastrangelo, E. F. Gherlone, G. Gastaldi

**Affiliations:** ^1^Dental School, Vita Salute University, 20132 Milan, Italy; ^2^Dental and Maxillofacial Surgery Unit, San Rocco Clinical Institute, Ome, 25050 Brescia, Italy

## Abstract

*Purpose.* Recently, the request of patients is changed in terms of not only esthetic but also previsualization therapy planning. The aim of this study is to evaluate a new 3D-CAD-CAM digital planning technique that uses a total digital smile process.* Materials and Methods.* Study participants included 28 adult dental patients, aged 19 to 53 years, with no oral, periodontal, or systemic diseases. For each patient, 3 intra- and extraoral pictures and intraoral digital impressions were taken. The digital images improved from the 2D Digital Smile System software and the scanner stereolithographic (STL) file was matched into the 3D-Digital Smile System to obtain a virtual previsualization of teeth and smile design. Then, the mockups were milled using a CAM system. Minimally invasive preparation was carried out on the enamel surface with the mockups as position guides.* Results.* The patients found both the digital smile design previsualization (64.3%) and the milling mockup test (85.7%) very effective.* Conclusions.* The new total 3D digital planning technique is a predictably and minimally invasive technique, allows easy diagnosis, and improves the communication with the patient and helps to reduce the working time and the errors usually associated with the classical prosthodontic manual step.

## 1. Introduction

In recent years, the concept of what makes a smile beautiful has changed significantly [1, 2]. Nowadays, patients expect complex functional rehabilitations that are esthetically appealing [2–5]. An important goal in prosthodontic is to use minimally invasive treatment to improve the appearance of the smile [3–6] as a way to valorize the entire image of the patient [7] while maintaining the health and function of teeth and soft tissue [8, 9].

Porcelain laminate veneers (PLVs), minimally invasive solutions to dental esthetic problems, have the most long-term success [7, 10–14]. There are a number of stages in rehabilitative dental treatment, from making the impression and developing the model to creating the diagnostic wax-up and to constructing the laboratory mockup. The planning associated with creating a mockup is a very important as it affects patients' understanding of the expected result [15, 16]. Whether the patient is happy with the overall treatment depends on how similar the prosthesis is to the mockup [17, 18]. The shape of the teeth, the adaptation of the prosthesis, and the size and the color of the new elements in relation to the soft tissue, lips, and the whole face are very important in the decision-making [19].

A large number of errors can occur at the various stages of the traditional prosthetic workflow, each stage requires a transfer of two-dimensional and three-dimensional (3D) data between operators. As computer-aided design and computer-aided manufacturing (CAD/CAM) and new materials are leading to a paradigm shift in what many practitioners regard as standard care for patients, a priority is to drastically reduce operator error [20].

The aim of this research was to evaluate new total 3D digital smile planning technique (3D-DSP) used in the previsualization stage prior to milling poly(methyl methacrylate) (PMMA) mockups in the process of creating PLVs using a CAD/CAM system.

## 2. Materials and Methods

Between September 2012 and July 2015, 28 patients (9 male and 19 female) aged 19 to 53 years (mean age of 36 years) took part in this study at the dental clinic at San Raffaele University, Milan, Italy. None of the patients had any oral, periodontal, or systemic diseases (Tables 1 and 2).

**Figure 1:**
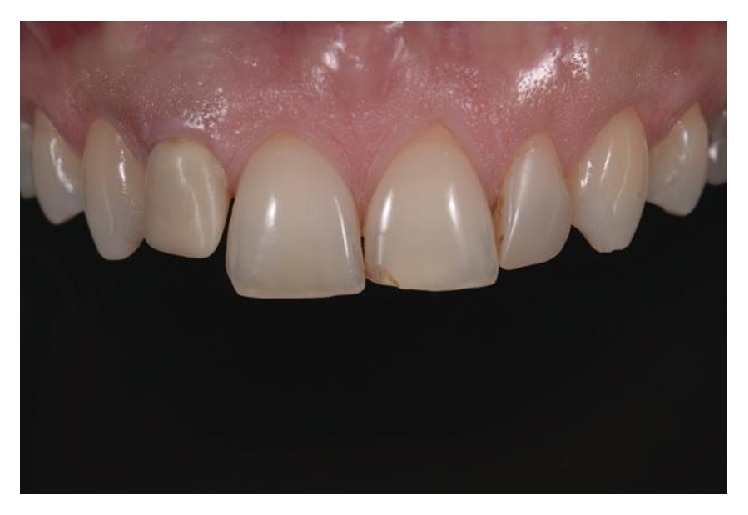
Initial clinical case intraoral photography.

**Figure 2:**
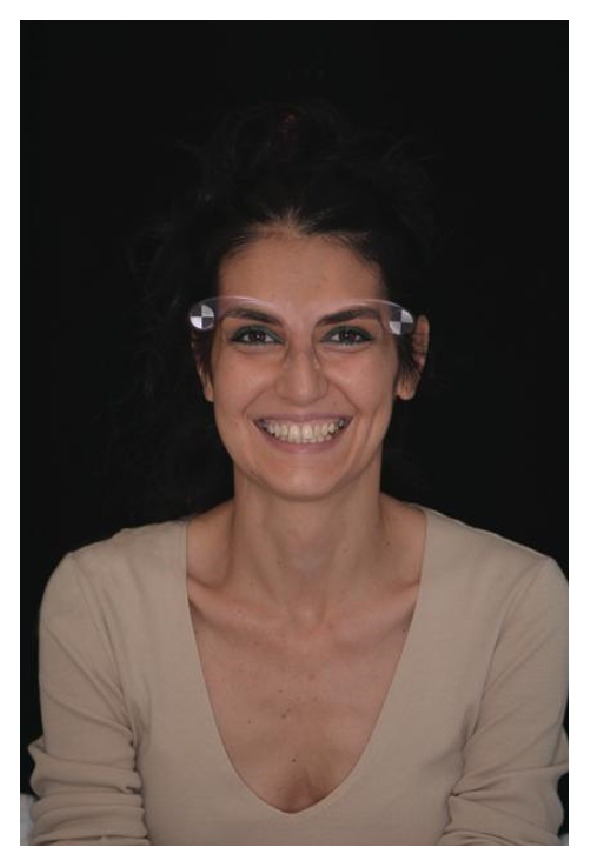
Initial clinical case extraoral photography.

**Figure 3:**
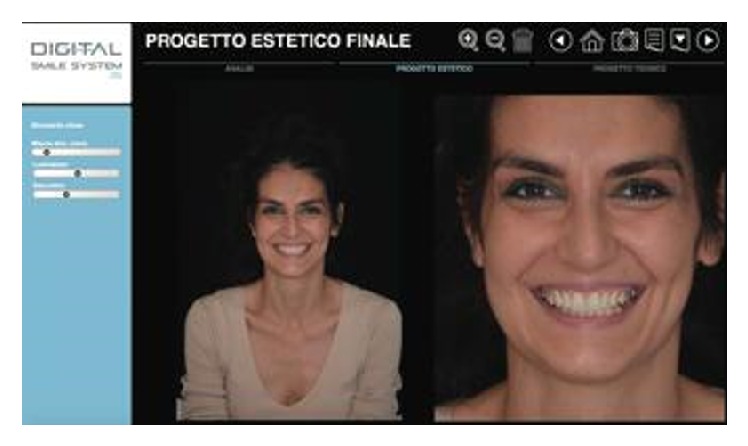
Digital smile design into Digital Smile System 2D.

**Figure 4:**
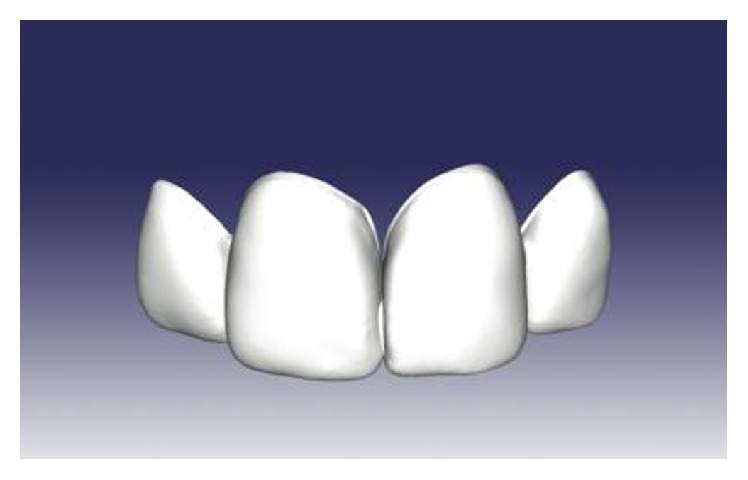
CAD design of the mockup.

**Figure 5:**
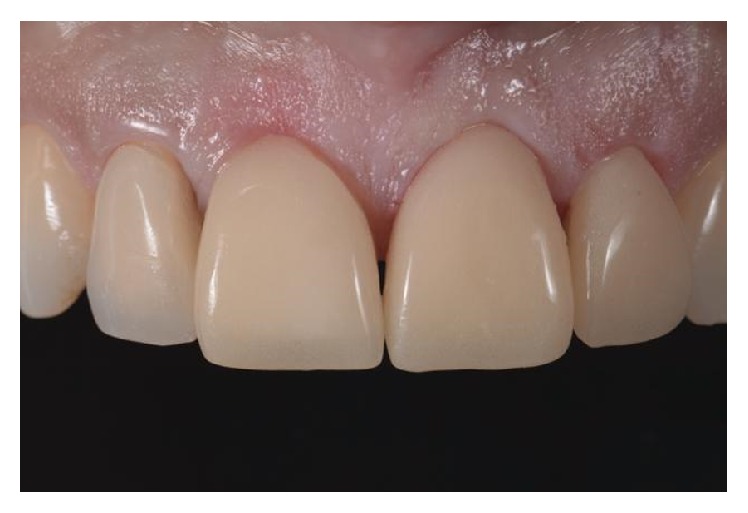
Intraoral evaluation of milling CAD-CAM mockup.

**Figure 6:**
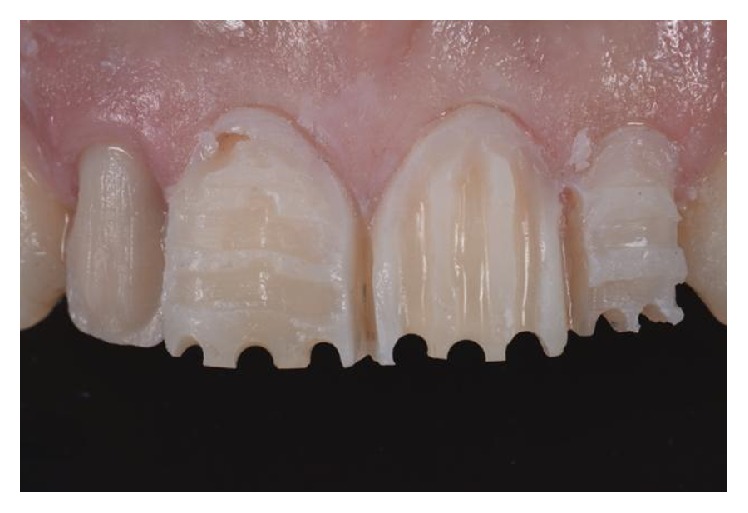
Mockup guide for teeth preparation.

**Figure 7:**
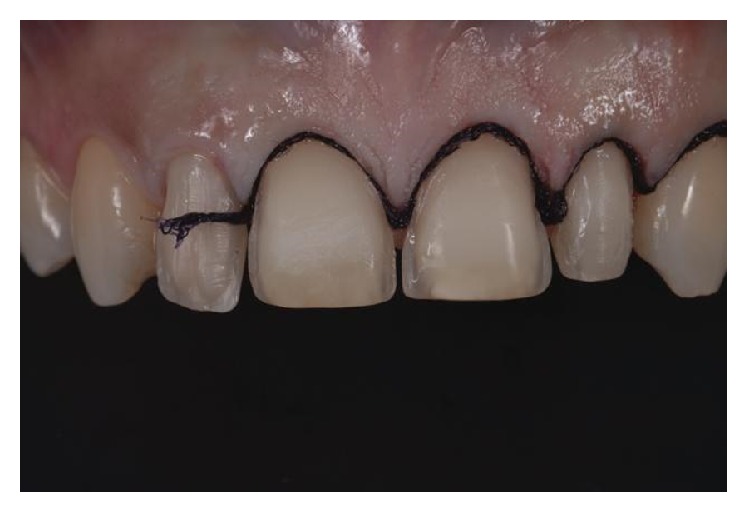
Double cord retraction technique.

**Figure 8:**
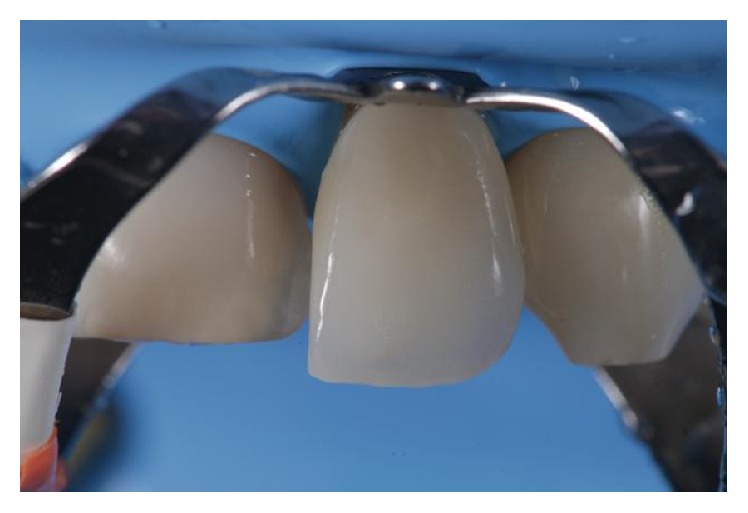
Adhesive cementation of the definitive veneers.

**Figure 9:**
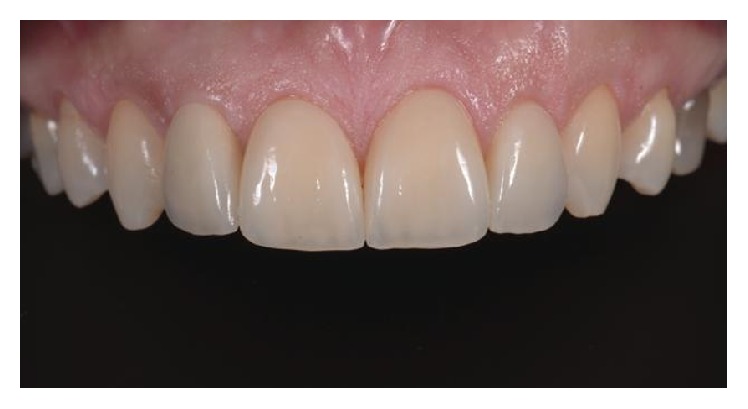
Final result: intraoral photography.

**Figure 10:**
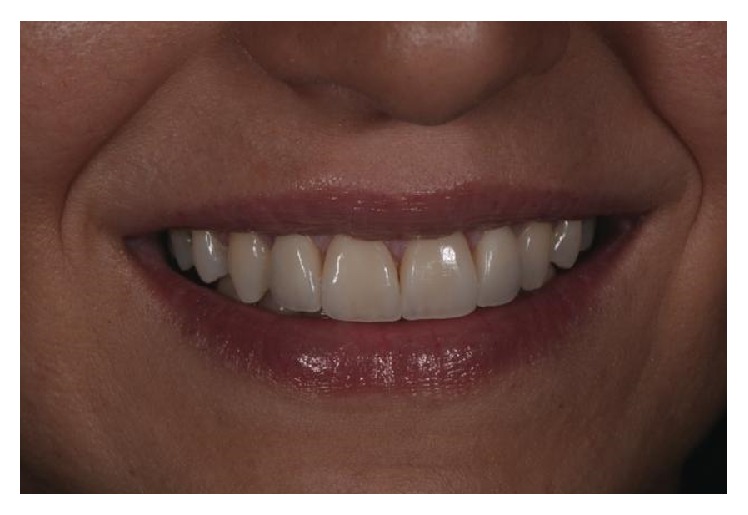
Final result: extraoral photography.

After radiological, phonetic, and static and dynamic occlusal evaluation, each patient had three intra- and extraoral digital images taken while wearing special eyewear (Digital Smile System Srl, Italy) (Figures 1 and 2). An intraoral scanner (Scanner 3D Progress, MHT, Italy) was used to get intraoral digital impressions of the maxilla and mandible arches in open and occlusal states. All the digital images, obtained from the processing of the pictures into the software 2D-Digital Smile System (Digital Smile System Srl, Italy) (Figure 3) and the STL file from the intraoral scans, were combined into the 3D-Digital Smile System (EGS Srl, Italy) to display the patient's teeth and, from this, a virtual design of the potential dental prosthesis was created. When the patient agreed to this virtual 3D view of their planned-for prosthetics, a PMMA mockup (Bredent Srl, Italy) was milled using a CAM system (Zirkonzahn Srl, Italy) (Figure 4). Each mockup was tested in the patient's oral cavity to make sure they would consent to the esthetic therapy and be satisfied with the end result (Figure 5). The newly milled mockups, cemented using spot-etch technique [21, 22], were used to guide the position of the prosthetics and maintain the margins on the enamel surface of the teeth [23–25] (Figure 6). The double cord techniques with the intraoral scanner (Scanner 3D Progress, MHT, Italy) was used to make all the definitive impression of the prepared teeth (Figure 7). The PLVs (IPS e.max System, Ivoclar Vivadent Srl, Italy) were produced using CAD/CAM technique (Zirkonzahn Srl, Italy). A total of 78 Variolink veneers (Ivoclar Vivadent Corp., Liechtenstein) and 30 Clearfil Esthetic Cement veneers (Kuraray America Inc., USA) were cemented onto vital teeth (Table 3) (Figure 8). Each patient had final intra- and extraoral digital images taken (Figures 9 and 10). Follow-up took place after 2 years.

## 3. Results

The preoperative patient parameters showed bruxism (22.2%), tooth trauma (14.8%), abrasion (11.2%), discoloration (22.2%), crowding (14.8%), diastema (7.4%), and caries (7.4%) (Table 4). The follow-up 2 years later revealed 1 total fracture, 2 sensitive teeth, and 1 gingival recession (0.9%). None of the 108 PLVs showed debonding, chipping, microleakage, discoloration, or secondary caries, and no root canal therapy was necessary (Table 5).

Patients responded to a questionnaire to determine their satisfaction with the digital smile design planning and the test in the form of the mockup. They graded both the planning and the test as effective, very effective, or ineffective. For the digital smile design previsualization, with visual analogical scale (VAS scale), 18 (64%) of patients found it very effective and 10 (36%) effective; 24 (86%) found the milling mockup very effective and 4 (14%) effective (Table 6).

## 4. Discussion

In all prosthodontic aesthetic treatment, the accurate design planning and the basic communication phase with the patient play a crucial role in the therapy. The best previsual means most widely used as a measure of explanation with a patient is the therapeutic planning, associated with the creation of a mockup [17, 19]. With contemporary digitalized techniques, it is possible to redesign a patient's smile [15, 16]. Effective previsualization followed by a mockup is the ideal way to explain changes to a patient and receive their approval [17–19]. Traditional “analogical techniques” are based on a planning process that involves radiological and clinical evaluation, intra- and extraoral photographic analysis, static and dynamic occlusal evaluation, and traditional impressions [21]. The more traditional techniques that use the free-hand “composite technique” before the wax-up do not evaluate the design of the smile [25, 26].

A secondary evolution of digital prosthetic planning is limited to bidimensional digital work flow [21] and requires, after digital smile design protocol, the stone model, the manual processing of a laboratory diagnostic wax-up, and the printing of the classic mockup in the patient's oral cavity through the use of silicone keys. In traditional planning techniques, the data transfer from virtual design to laboratory is difficult and potentially full of errors because it uses a manual process to obtain the computer design of canine zenith lines for the laboratory stone model [21]. This manual process is necessary to transfer all the measurements of the teeth to the new smile project design. Another difficult and unpredictable process is the mockup printing in the patient's oral cavity with a silicone mask (made on a wax-up) [10–21]. Our new planning technique allows a new totally digital and CAD-CAM process, from the initial photo shoot to CAD/CAM-milling mockup, to reduce the errors usually associated with the classical manual steps and to improve the accuracy of the prosthetic procedure. All digital data transfer from the clinical 3D planning to the laboratory CAD/CAM process is simpler, faster, and more predictable. However, having photographs plays a crucial role: the patient-approved virtual smile is used to guide the final design of the teeth, which are usually made with the CAD/CAM process.

## 5. Conclusions

A 2-year follow-up of prosthetic PLVs created using the new total digital smile planning technique in vital teeth in the esthetic zone showed that it is possible to obtain excellent results in both functional and esthetic rehabilitation and high patient satisfaction. The new procedure also reduces the amount of time spent in the clinic and laboratory, increases the predictability of data matching to build CAD/CAM-milling mockups, reduces trauma caused by handling hard dental tissues, and improves accuracy and reproducibility of the final mockup. The total new digital smile planning technique is minimally invasive and facilitates diagnosis, improves communication with the patient, reduces processing times, and increases predictability of the results with very little discomfort and very high esthetic final results. The present study has limits, such as the limited number of patients enrolled: further studies on a larger sample of patients are therefore needed to confirm our present results.

## Figures and Tables

**Table 1:** Number of the patient veneers restorations.

	Number of treated patients	%
Males	9	32.2
Female	19	67.8

Total	28	100

**Table 2:** Distribution of porcelain laminate veneers according to location.

		Veneers	
		Number (*n*)	Percentage (%)
Maxilla	Anterior	54	50
	Posterior	30	27.8
	Total	84	77.8

Mandible	Anterior	10	9.3
	Posterior	14	12.9
	Total	24	22.2

Total		108	100

**Table 3:** Distribution of PLVs according to bonding material.

Veneers (CAD-CAM)	108	100
Variolink veneers (Ivoclar Vivadent)	78	72.2
Clearfil esthetic cement (Kuraray)	30	27.8

Total	108	100

**Table 4:** Preoperative parameters.

	Patients		Teeth	
	Number (*n*)	Percentage (%)	Number (*n*)	Percentage (%)
Trauma	2	7.2	16	14.8
Bruxism	4	14.3	24	22.2
Abrasion	6	21.4	12	11.2
Discoloration	6	21.4	24	22.2
Crowding	4	14.3	16	14.8
Diastema	2	7.1	8	7.4
Caries	4	14.3	8	7.4

Total	28	100	108	100

**Table 5:** Distribution of failures according to preparation design.

	Among 28 patients	%	Among 108 veneers	%
Fracture	1	3.6	1	0.9
Chipping	0	0	0	0
Debonding	0	0	0	0
Microleakage	0	0	0	0
Secondary caries	0	0	0	0
Sensitivity	2	7.1	2	1.8
Root canal treatment	0	0	0	0
Gingival recession	1	3.6	1	0.9
Discoloration	0	0	0	0

**Table 6:** Appreciation of the previsualization with VAS scale.

Test with smile design	Very effective	18	64.3%
	Effective	10	35.7%
	Ineffective	0	0%
	Total	28	100%

Test with mockup	Very effective	24	85.7%
	Effective	4	14.3%
	Ineffective	0	0%
	Total	28	100%
